# Editorial: Hypertensive disorders of pregnancy and the cardiovascular system: Causes, consequences, prevention and treatment

**DOI:** 10.3389/fcvm.2023.1179616

**Published:** 2023-04-03

**Authors:** Inge Dierickx, Robert-Jan Alers, Rossana Orabona, Edoardo Sciatti, Marc Spaanderman, Federico Prefumo, Chahinda Ghossein-Doha

**Affiliations:** ^1^ Department of Obstetrics and Gynaecology, Sint Lucas Ziekenhuis, Gent, Belgium; ^2^ Department of Physiology, Hasselt University, Hasselt, Belgium; ^3^ Department of Obstetrics and Gynecology, Maastricht University Medical Center+ (MUMC+), Maastricht, Netherlands; ^4^ GROW, School for Oncology and Developmental Biology, Maastricht University, Maastricht, Netherlands; ^5^ Obstetrics and Gynecology Unit, ASST Spedali Civili, Brescia, Italy; ^6^ Cardiology Unit 1, ASST Papa Giovanni XXIII, Bergamo, Italy; ^7^ Department of Obstetrics and Gynecology, Radboud University Medical Center, Nijmegen, Netherlands; ^8^ Obstetrics and Gynecology Unit, IRCCS Istituto Giannina Gaslini, Genova, Italy; ^9^ Department of Cardiology, Maastricht University Medical Center+ (MUMC+), Maastricht, Netherlands; ^10^ CARIM, School for Cardiovascular Diseases, Maastricht University, Maastricht, Netherlands

**Keywords:** hypertension, cardiovascular system, pregnancy, causes, consequences, prevention, treatment

**Editorial on the Research Topic** Editorial: Hypertensive disorders of pregnancy and the cardiovascular system: Causes, consequences, prevention and therapy

## Introduction

Hypertension occurs in one in ten pregnancies and may progress into preeclampsia when signs of organ damage emerge, and eclampsia when seizures ensue (1). Unsurprisingly, hypertension puts the pregnant mother and her child at risk of severe morbidity and death. Over the past years incidence rates of gestational hypertensive disorders have risen despite strenuous efforts of researchers and medical professionals (1). After gestational hypertension, women are at risk of developing cardiovascular and cardiometabolic disease, including diabetes, atherosclerosis, and chronic hypertension later in life. Proper insights into its causes, consequences, prevention and treatment are imperative in our efforts to improve future women's health.

This special issue of “*Hypertensive disorders of pregnancy and the cardiovascular system*” presents substantial scientific work aimed at underlying mechanisms, risks factors, and sequelae of preeclampsia. Important steps towards a better understanding of gestational hypertension and identifying pregnancies at risk of severe morbidity are discussed and a new study protocol is introduced.

## Causes

Preeclampsia is a multifaceted syndrome with numerous hypotheses regarding its aetiology and pathophysiology. A generally accepted consensus is yet to be obtained and the studies presented here aim to gain a better understanding of its causes from genetic, metabolic, and hemodynamic perspectives.

Disturbances of lipid metabolism precede the onset of preeclampsia. Both overweight and underweight link to poor maternal and neonatal outcomes. Hu et al. observed lowest risks of disease when the BMI is between 19 and 23 preconceptionally, which is a narrower range than the common notion of healthy BMI between 18.5 and 25. Interestingly, risks of gestational hypertension already increase from BMI values 20 and up.

As preeclampsia has the tendency to run in families, a genetic origin of disturbed lipid metabolism was studied by Liu et al. A specific enzyme involved in lipid metabolism, APOBEC3A, is overexpressed in preeclampsia. Its hypothetical contribution to preeclampsia is further substantiated by the observation of disturbed lipid metabolism and a preeclampsia-like phenotype in an animal model of pregnant APOBEC3A-overexpressing mice. Complications associated with preeclampsia, such as renal impediments, may also have genetic origins. The Chinese Han population, for instance, has a relatively high incidence of hypertensive renal damage and impaired renal function during preeclampsia. In this population, Yun et al. discovered that the C677T gene mutation of the MTHFR-gene that affects methionine metabolism constitutes an independent risk factor for impaired renal function during preeclampsia.

Adequate maternal cardiovascular adaptation is key to uncomplicated course of pregnancy. Cardiovascular maladaptation can result in preeclampsia and may originate from a maternal prothrombotic state, increased vascular tone, or reduced volume circulation as is discussed in the works of Godtfredsen et al., Dierickx et al. and Gyselaers and Lees Components of the contact activation system are not only involved with coagulation and inflammation processes, but also with the regulation of placental function. Godtfredsen et al. used new methods focusing on the dynamic interactions and total capacity of the contact activating system in blood. They found that several components, specifically kallikrein generating capacity, prekallikrein and cleaved H-kininogen, are reduced in women with preeclampsia.

Findings supporting vascular maladaptation during preeclampsia are presented by Dierickx et al. Their combined Doppler-ECG assessment of the internal jugular veins demonstrated a lower venous pulse transit time at internal jugular veins in early-onset preeclampsia, suggesting that there is an increased venous vascular tone at supracardial level.

Gyselaers and Lees review existing evidence on the association between maternal intravascular filling state and fetal growth (Gyselaers and Lees). Previous studies in pregnant rodents showed that low plasma volume predisposes to poor fetal growth and beneficial effects are seen after enhancing plasma volume. A low intravascular volume combined with a high vascular resistance is suggested to cause hypertension in women with preeclampsia and fetal growth restriction. This review is an invitation to further investigate whether elevating the maternal intravascular volume in pregnant women with fetal growth retardation and preeclampsia may be a potential target for prevention and management.

Microvasculature changes also occur during pregnancy as is demonstrated with nailfold video capillaroscopy by Thevissen et al. The capillary bed of women with a low-risk cardiovascular profile differs in density and diameters compared to women with a high-risk cardiovascular profile. Furthermore, the capillary bed remains unchanged during gestation in low-risk women, whereas distinct differences exist in women with a high-risk cardiovascular profile and women with preeclampsia separately. Future studies should investigate whether these differences can be used to predict onset of preeclampsia.

## Consequences

Pregnancy may be viewed upon as cardiometabolic stress test. As supported by the meta-analysis of Xu et al. pregnancy-induced hypertension and preeclampsia may be the first early signs flagging women who are especially prone to develop chronic hypertension later in life. These risks differ based on ethnic and racial origins as is clearly reviewed by Burger et al. Hypertensive diseases of pregnancy are associated consistently with increased cardiovascular disease risk across racial and ethnic groups. However, it is remarkable that the risk of gestational hypertension in non-White women is lower or similar compared to that found in non-Hispanic White women, while chronic hypertension, (superimposed) preeclampsia, and eclampsia risks are increased among most non-White populations.

From a logical perspective, strict blood pressure control seems especially important in women with a history of gestational hypertension to prevent chronic hypertension and its associated complications later in life. Increasing blood pressure, for example, is associated with a higher risk of mortality in patients with aortic dissection as shown by Wu et al. Interestingly, blood pressure is best interpreted dynamically over time because decelerating drops in pressure are associated with better outcomes while accelerating rises had poor prognosis. Nonetheless, low blood pressure can be harmful as well. In an intensive care unit setting, Shao et al. demonstrate that the mortality risk of patients with atrial fibrillation increases with hypertension, but also increase with pressures below 110 mmHg systolic blood pressure, below 55 mmHg diastolic blood pressure, and below 70 mmHg mean arterial pressure.

One interesting hypothesis is that women after preeclampsia are older in a vascular sense compared to women of equal biological age with a history of normotensive pregnancies. From a functional perspective, this seems not the case as Jansen et al. show that brachial artery flow-mediated dilation and sublingually administered nitroglycerine-mediated dilation decrease with age at comparable rates in young- to middle-aged women with a history of preeclampsia versus a history of normotensive pregnancy. These findings suggest that an increased cardiovascular risk in the first decades after preeclampsia does not originate from an accelerated decline in endothelial function. Perhaps a different site, the microcirculation, ages more rapidly or another mode of endothelial action is involved, such as hemostatic and inflammatory related integrity. These hypotheses remain subjects for further investigation.

## Prevention and treatment

Once preeclampsia develops, it progressively worsens. There is currently no treatment other than terminating the pregnancy once maternal or fetal life is in serious danger (2). Therefore, many researchers try to find ways to prevent the onset of preeclampsia. As described above, pregestational low intravascular volume and preeclampsia associate with fetal growth restriction. Gyselaers and Lees propose physical exercise before pregnancy as an attempt to increase maternal intravascular volume and reduce the risks of fetal growth restriction and early-onset preeclampsia. The beneficial effects of an increased filling state have been shown previously in pregnant rodents although its effectiveness is yet to be demonstrated in humans.

To reduce the risk of preeclampsia onset, administration of low-dose aspirin is commonly advised for women at risk (3). Because this recommendation is based on studies with singleton pregnancies, its effectiveness in twin pregnancies was studied by Zhou et al. Perhaps due to poor compliance, no risk reduction is observed. Their findings highlight the difficulties faced when assessing risk reducing regimens.

Means of preventing disease progression are still highly desired when preeclampsia strikes. In early onset preeclampsia expectant management is usually attempted to decrease the likelihood of fetal morbidity. Women with mild hypertension should be induced to deliver after 37 weeks of gestation (4). However, the timing of hospitalization, corticosteroids, and delivery remain a clinical challenge in case of severe hypertension. Villalain et al. developed two models with promising clinical potential using clinical characteristics such as BMI, gestational age, and fetal weight, and biomarkers placental growth factor (PlGF) and soluble fms-like tyrosine kinase-1 (sFlt-1). Their first model predicts the need to deliver within 7 days in women with early-onset preeclampsia and their second model the development of HELLP or placental abruption with 76% and 90% predictive values, respectively. These models potentially aid decision making regarding corticosteroid administration and expectant management.

Tanaka et al. further explored the use of biomarkers in timing delivery of women with early onset preeclampsia. Delivery within one week was predicted using sFlt-1 with a 67% positive predictive value. However, the study population was small and larger cohorts are necessary to determine the clinical feasibility of sFlt-1 in predicting time of delivery. Ratnik et al. also studied the use of biomarkers. They discovered that blood samples should be obtained between 10 and 12 weeks of gestation to predict preeclampsia with 80% specificity and 88% accuracy using a panel of biomarkers (PTX3, sFlt-1, and ADAM12) in combination with the subject's parity and gestational age at blood sampling. In future studies, their model as a first trimester screening tool for hypertensive gestational disease should be validated to allow careful early intervention and monitoring of individuals at-risk.

The use of biomarkers is costly which might prevent their feasibility as a screening instrument. To overcome this hurdle, Trilla et al. present a prospective, multicentric, cohort study protocol that aims to screen for preeclampsia in a two-step sequential screening model. The first step aims to distinguish women with low-risk from those with an intermediate or high risk of preeclampsia based on their medical history, mean blood pressure, Pregnancy-Associated Plasma Protein A (PAPP-A), and pulsatility index of the uterine arteries. In the second step, PlGF and sFLT-1 values are determined in women with an intermediate and high risk to allow reclassification into low-risk and high-risk groups. Predictive and preventive capacity of this two-step sequential model and the financial cost will be compared to the universal screening system.

All together, we hope you will enjoy reading these articles in this issue of Frontiers in Cardiovascular Medicine.
